# Killer Immunoglobulin-like Receptors (KIR) haplogroups A and B track with Natural Killer Cells and Cytokine Profile in Aged Subjects: Observations from Octo/Nonagenarians in the Belfast Elderly Longitudinal Free-living Aging STudy (BELFAST)

**DOI:** 10.1186/1742-4933-10-35

**Published:** 2013-08-19

**Authors:** Irene Maeve Rea, Lynn D Maxwell, Susan E McNerlan, H Denis Alexander, Martin D Curran, Derek Middleton, Owen A Ross

**Affiliations:** 1 School of Medicine, Dentistry and Biomedical Science Queens University, Belfast, UK; 2 Immunology and Microbiology Laboratory, Belfast Health and Social Care Trust, Belfast, UK; 3 Cytogenetics Laboratory, Belfast Health and Social Care Trust, Belfast, UK; 4 School of Biomedical Science, University of Ulster, Coleraine, UK; 5 Molecular Diagnostic Microbiology Section, Health Protection Agency, Addenbrookes Hospital, Cambridge, UK; 6 Transplantation Centre, Liverpool, UK; 7 Mayo Clinic Jacksonville, Jacksonville, FL, USA

**Keywords:** Ageing, BELFAST octo/nonagenarians, KIR A and B haplotypes, Cytokines, IL-6, IL-12, IL-12p40, IL-10, Active TGF-β, sIL-2R, TNF-α

## Abstract

**Background:**

Natural Killer Cells (NK) play an important role in detection and elimination of virus-infected, damaged or cancer cells. NK cell function is guided by expression of Killer Immunoglobulin-like Receptors (KIRs) and contributed to by the cytokine milieu. KIR molecules are grouped on NK cells into stimulatory and inhibitory KIR haplotypes A and B, through which NKs sense and tolerate HLA self-antigens or up-regulate the NK-cytotoxic response to cells with altered HLA self-antigens, damaged by viruses or tumours. We have previously described increased numbers of NK and NK-related subsets in association with sIL-2R cytokine serum levels in BELFAST octo/nonagenarians. We hypothesised that changes in KIR A and B haplotype gene frequencies could explain the increased cytokine profiles and NK compartments previously described in Belfast Elderly Longitudinal Free-living Aging STudy (BELFAST) octo/nonagenarians, who show evidence of ageing well.

**Results:**

In the BELFAST study, 24% of octo/nonagenarians carried the KIR A haplotype and 76% KIR B haplotype with no differences for KIR A haplogroup frequency between male or female subjects (23% v 24%; p=0.88) or for KIR B haplogroup (77% v 76%; p=0.99). Octo/nonagenarian KIR A haplotype carriers showed increased NK numbers and percentage compared to Group B KIR subjects (p=0.003; p=0.016 respectively). There were no KIR A/ B haplogroup-associated changes for related CD57+CD8 (^high or low^) subsets. Using logistic regression, KIR B carriers were predicted to have higher IL-12 cytokine levels compared to KIR A carriers by about 3% (OR 1.03, confidence limits CI 0.99–1.09; p=0.027) and 14% higher levels for TGF-β (active), a cytokine with an anti-inflammatory role, (OR 1.14, confidence limits CI 0.99–1.09; p=0.002).

**Conclusion:**

In this observational study, BELFAST octo/nonagenarians carrying KIR A haplotype showed higher NK cell numbers and percentage compared to KIR B carriers. Conversely, KIR B haplotype carriers, with genes encoding for activating KIRs, showed a tendency for higher serum pro-inflammatory cytokines compared to KIR A carriers. While the findings in this study should be considered exploratory they may serve to stimulate debate about the immune signatures of those who appear to age slowly and who represent a model for good quality survivor-hood.

## Background

Natural Killer cell populations, their KIR receptor complexes and associated cytokine profiles which both generate and drive their responsiveness, are highly effective collaborators in patrolling, controlling and protecting our immune landscape thorough out life. Their roles and interactions are interdependent and their profiles are of interest since they are likely to be important in maintaining immune integrity in people who live successfully into their 90s and fit the criteria of the Perl ‘escaper’ model of successful ageing [1].

NK cells bridge between the innate and adaptive immune responses. They are not homogeneous and subsets can be identified in various ways; NK cells have no T-cell receptor CD3, but are usually positive for CD16, CD56 and frequently CD57(HNK-1). CD56 intensity of expression (dim or bright) on NK cells also determines functional differences in cytotoxicity and cytokine production [2,3], with CD56^bright^ NK cells being described as the “cytokine responsive” NK cell subset which does not require “licensing” by host MHC-I molecules and which express low levels of perforin [4]. CD8 antigen is present on 30-40% of NK cells [5,6] and identifies another group of cytotoxic cells which may also co-exist with CD57 [7]. CD8+(^high^)CD57+ cells demonstrate high cytotoxic potential associated with perforin, granzyme and adhesion molecule expression, compared to the CD8+(^low^)CD57 subset [8-11]. In healthy people, CD57 antigen is expressed by a minority of CD8+T lymphocytes but increased numbers of CD8+ CD57+ cells are found associated with chronic inflammation, cancer status and with increased age [12]. The level of expression of CD8 on NK subsets allows separation of the NK and cytolytic T-lymphocyte (CTL) components of the CD57+ subset.

NK cells and associated subsets express a combination of receptors which provide them with a broad capability for eliminating virus- or tumour- damaged cells. The Killer Inhibitory Receptors (KIR) NK genes are grouped in 15–17 genes (dependent on nomenclature) and map to chromosome 19q13.4 in a discrete area of 150kb within the leucocyte receptor complex. They have been classified into 4 major groups KIR2DS, 3DS, 2DL, 3DL on the basis of the number of extracellular domains (2 or 3) and whether they have long (L) usually inhibitory or short (S) usually activatory, intracellular cytoplasmic receptor-linking signalling tails. KIRs act by binding to HLA-ligands and provide NK cells with specialised killer functions [13-15] mediated through activating and inhibiting signals [16]. NK cells do not destroy cells which express normal levels of surface MHC class-I because inhibitory signals dominate, whereas cells whose surface MHC Class-I molecules are damaged by viruses or tumours are primed for destruction through activator KIR signals [17]. The absence of a single MHC-I allele, common in cancer cells, sensitizes the cell to NK cell cytotoxicity [18,19]. This role is exploited in medical oncology and bone marrow transplantation where NK cells kill host/donor lympho-haemopoietic cells which by expressing different HLA-Class I molecule/s, mismatch and trigger NK inhibitory receptors [20].

At the population level, KIR genes can be separated into two major haplotypes-A and B. Both haplotypes have 3 common conserved framework genes - KIR3DL3, KIR3DP1 (P refers to pseudogene), and KIR3DL2 that are separated by a variable number and type of KIR genes depending on A or B haplotype [21]. The simpler group-A KIR haplotype is generally non variable and comprises a fixed gene content of inhibitory genes-KIR 2DL1, 2DL3 and 3DL1 *with 2DS4 the single activating gene*. Group-B KIR haplotype contains a variable gene combination but tends to encode more activating KIRs [22] and normally does not include 2DS4 [23]. The framework genes are generally held to be KIR 3DL3, 3DP1, 3DL2 and 2DL4 with KIR2DL4 being a specific KIR gene which has a long cytoplasmic tail but which mediates both inhibitory and activatory signals.

The function of NK cells contributing to and maintaining immune integrity is guided by KIRs and contributed to by the cytokine milieu. The profile of A and B KIR genotypes is increasingly being recognised as individual to people and populations and driven by pathogen experience and cytokines [21]. We previously described increased NK and NK-related subsets and associated sIL-2R serum levels in octo/nonagenarians in the BELFAST study [24,25], who show evidence of ageing well [26].

In this observational study, we further enquired whether KIR A and B haplotype frequencies were changed in the BELFAST octo/nonagenarian cohort who met the Perl ‘*escaper*’ concept [1] and whether any change might explain the increases in the NK cell subsets and cytokine profiles previously noted in the BELFAST aged cohort.

## Results

### Subject phenotype characteristics

#### Subjects

In the BELFAST study, 24% of octo/nonagenarians carried the KIR A and 76% KIR Group B KIR haplotype with no differences for KIR A haplogroup carriage between male or female subjects (23% v 24%; p=0.88) or for KIR B haplogroup (77% v 76%; p=0.99). Subject characteristics categorised by KIR A and B haplotypes are described in Table 1. Mean age was 88.2[SD 5.1] years with no difference between KIR A and KIR B haplotype (p=0.07) carriers. There was also no significant difference for anthropometric variables-waist, body mass index (BMI), and triceps skin fold thickness (TSF) for comparison by KIR A and B haplotypes though weight showed significantly higher values for KIR haplotype A carriers (p=0.02). Using regression analysis, there was a significant association between BMI with NK cells for KIR B haplotype carriers separately (p=0.05) but not for the smaller number of KIR A carriers (p=0.58). There were no significant differences for Free Thyroxine FT4 and the biochemical variables urea, glucose and folate between KIR A and KIR B haplogroup carriers. Serum copper was significantly lower in KIR B haplogroup carriers (p=0.02) but serum selenium and zinc showed no differences between KIR groups.

**Table 1:** Phenotypic characteristics of Octo/Nonagenarian subjects from the Belfast Elderly Longitudinal Free-Living Ageing STudy (BELFAST) categorised by KIR A and KIR B Haplotypes with comparisions by Student’s t-test or Mann Whitney U test

**Variable**	**KIR A**	**KIR B**	**t-test or *mwuω***
**Age yrs**	**87.1**±6.0(21)	**89.3**±4.1(56)	0.07
**Sex**	8M/13F	17M/39F	0.59
**Weight kg**	**66.1**±13.6(20)	**58.6**±11.4(53)	0.02*
**Waist cm**	**90.4**±15.8(18)	**86.2**±13.4(53)	0.28
**BMI kg/m**^**2**^	**24.5**±4.3(18)	**23.5**±4.0(53)	0.40
**TSF mm**	**15.4**±7.1(17)	**12.8**±6.3(51)	0.16
**FT4 ug/dl**	**15.2**±6.2(19)	**15.6**±3.6(52)	0.69
**Urea umol/l**	***8.8*** *[7–11.2](7)*	**7.7***[4-12](15)*	*0.38* ***ω***
**Glucose umol/l**	**5.4***[3.7–7.3] (7)*	**5.7***[4.2–8.9](15)*	*0.72* ***ω***
**Folate ug/l**	**4.3***[1.2–6.2](7)]*	**3.8***[2.1–9.1](13)*	*0.63* ***ω***
**Selenium umol/l**	**0.81**±0.18(19)	**0.80**±0.28(51)	0.83
**Zinc umol/l**	**12.8**±1.9(20)	**12.4**±1.9(55)	0.43
**Copper umol/l**	**20.2**±2.6(20)	**18.4**±3.1(55)	0.02*

*BMI* body mass index, *TSF* triceps skin fold thickness, *FT4* Free thyroxine index,.

Mean ±Standard Deviation, Subject nos (), Students t-test t-test, *p<0.05 is significant.

Median and range, Subject nos (), Mann Whitney-U mwu ***ω***.

### NK and associated subsets categorised by KIR A and B haplogroups for BELFAST octo/nonagenarians

#### Natural Killer cell CD3-CD16+CD56+, CD3-CD16+CD56+% and KIR A and B haplotypes

The mean white cell count across the BELFAST octo/nonagenarian group was 7.0×10^6^ (SD2.0) with no difference between KIR A and B haplotype carriers (p=0.76). The mean NK CD3-16+56+ cell count for BELFAST octo/nonagenarians was 0.35 ×10^6^ with a significantly larger number of NK cells (p=0.003) associated with subjects carrying the KIR A phenotype (0.48×10^6^ [SD 0.31]) compared to B phenotype (0.33×10^6^ [SD 0.18]). A similar pattern was seen for percentage of NK cells with significantly higher values (p=0.016) associated with KIR A compared to KIR B phenotypes (23.4% [SD 9.5]) and (18.4% [SD 9.9] respectively Table 2. Although a similar trend for higher values was present for KIR A haplogroup for each sex separately, there were no significant sex-related differences across the NK cell counts (p=0.95) or percentages (p=0.86)[data not shown].

**Table 2:** Numbers and percentages of NK and NK-related subsets categorised by KIR A and B Haplotypes for Octo/Nonagenarians from the Belfast Elderly Longitudinal Free-Living Ageing STudy (BELFAST) with comparisons by Students t-test or Mann–Whitney U

	**KIR A**	**KIR B**	**t-test/mwu *ω***
**Wcc x10**^**6**^	**6.9** ±1.6(19)	**7.1**±2.5(54)	0.76
**NK CD3-16+56+ct**	**0.48**±0.31(19)	**0.33**±0.18(46)	0.003*
**NK CD3-16+56+%**	**23.4**±9.5(21)	**18.4**±9.9(48)	0.016*
**CD57+CD8+ct**	**0.24***[0.7–1.6](7)*	**0.30***[0.3–0.8](22)*	*0.58ω*
**CD57+CD8+%**	**13***[5–25](7)*	**18***[3–45](22)*	*0.28ω*
**CD57+CD8(**^**high**^**)+ct**	**0.13***[0.03–0.17](7)*	**0.16***[0.01–0.53](19)*	*0.58ω*
**CD57+ CD8(**^**high**^**)+%**	**5.0***[2–6](7)*	**8.0***[1–21](19)*	*0.54ω*
**CD57+ CD8(**^**low**^**)+ct**	**0.16***[0.03–0.25](7)*	**0.17***[0.09–0.19](19)*	*0.58ω*
**CD57+ CD8(**^**low**^**)+%**	**6.0***[8–15](7)*	**11.0***[1–40](19)*	*0.37ω*

*Wcc* white cell count.

Mean ± Standard Deviation, Subject nos (), Students t-test t-test, *p<0.05 is significant.

Median and range, Subject nos (), Mann Whitney-U mwu ***ω***.

### NK-related subsets CD57+CD8+, CD57+CD8(^High^) and CD57+CD8(^low^) cells and KIR A and B haplotypes

The counts and percentages for the NK-related CD57+CD8(^High^)+ and CD57+CD8(^low^)+ subsets are included for completeness with medians and ranges in Table 2. There are no differences across KIR A and B haplogroups by non parametric comparison, but numbers are small and values should be considered as descriptive only.

### Cytokine profiles and KIR A and B haplotype carriers

**Figure 1:**
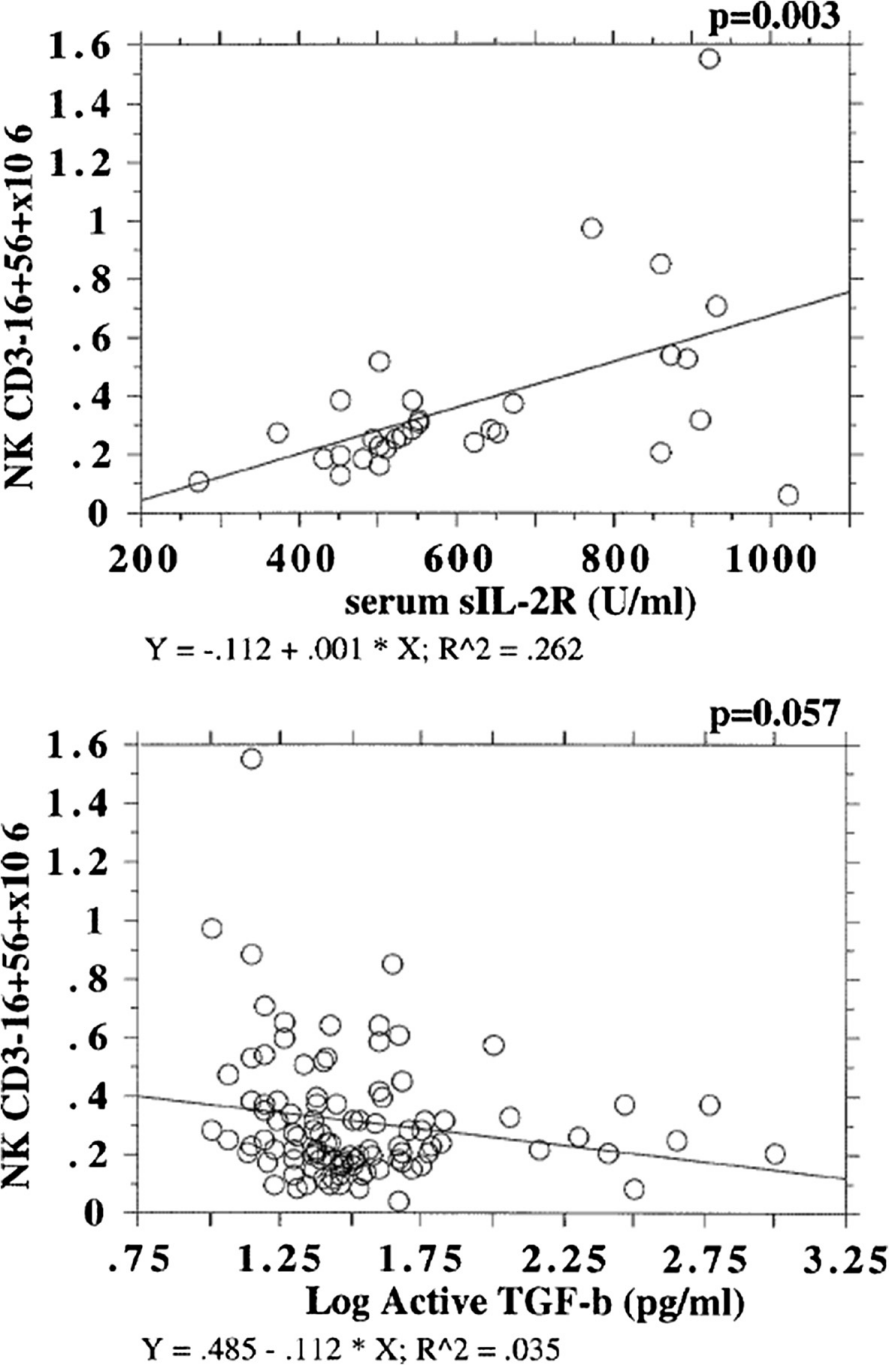
**Regression Scatterplots and Regression lines for Serum Cytokines sIL-2R U/ml and TGF-β pg/ml with Natural Killer cells x10**^**6 **^**(NK) for Octo/Nonagenarians from the Belfast Elderly Longitudinal Free-Living Ageing STudy (BELFAST) with associated p values.**

Simple regression analysis was used to assess any association between pro-and anti-inflammatory cytokines and NK subset numbers or percentage, which might suggest a causal relationship. There was a weak positive association between increasing levels of the pro-inflammatory cytokines sIL-2R, IFN-γ, and IL-12 with increasing NK numbers in the range of 3% and 14%. Conversely the anti-inflammatory cytokine TGF-β (Log active) showed a trend for a negative association with NK numbers with no change for IL-10 (Figure 1).

The cytokine profile for the measured **pro-and anti-inflammatory** cytokines, categorised for KIR A and B haplotype carriage is shown in Table 3. Medians, ranges and p values for KIR A and B haplogroup comparisons by non parametric Mann Whitney-U (mwu) analysis, show suggestive p values for IL-12 and TGF-β group members, but numbers are small and results should be considered as exploratory rather than definitive. Fold differences also estimated in Table 3, show a consistent trend for KIR B haplotype carriers to show higher median values for the varying cytokines by increases of between 1.2-4.0 fold.

**Table 3:** Pro- and anti-inflammatory cytokines categorised by KIR A and B haplogroups for Octo/Nonagenarians from the Belfast Elderly Longitudinal Free-Living Ageing STudy (BELFAST) with comparisons by Mann–Whitney U and Fold difference between KIR A and B cytokine levels

**Cytokine**	**KIR A**	**KIR B**	**mw-u** ***ω***	**Fold diff (KIR A v KIR B)**
**sIL-2R**	**640***[500–770](3)*	***865*** *[540–1600](16)*	*0.09ω*	1.35
**IFN-γ**	**1.5***[1.1–3.9](6)*	**1.8***[0.7–14.4](14)*	*0.51ω*	1.2
**TNF-a**	**25***[16–43](12)*	**26***[8–56](34)*	*0.96ω*	1.0
**sIL-6R**	**47.6***[24–55](5)*	**52.8***[25–104](16)*	*0.26ω*	1.11
**IL-6**	**6***[5–40](12)*	**9***[1–36](34)*	*0.69ω*	1.5
**IL-10**	**1.0***[0–6.5](5)*	**2.0***[0–4](16)*	*0.28ω*	2.0
**IL-12**	**97***[69–122](6)*	**285***[89–539](14)*	0.003*ω	2.9
**IL-12p70**	**8.2***[4–13](6)*	**5.4***[3–87](14)*	*0.17ω*	0.65
**IL-12p40**	***104***[16-160](6)	**280**[13-430](14)	0.002* ***ω***	2.7
**12p40/p70**	**10.2***[6.2–22.5](6)*	**49.4***[5–79](14)*	0.01*ω	4.8
**TGF-β**	**33.7***[0–50](10)*	**32.5***[17.3–52](34)*	**0.52** ***ω***	0.96
**1/logTGF-β**	**0.83***[0.6–1.0](10)*	**0.70***[0.33–0.94](34)*	0.02*ω	0.84
**TGF-β(active)**	**15.6***[0–50](10)*	**26.9***[11–1000](33)*	0.009*ω	1.7

Median, range [], subject no (), Mann Whitney-U test, mwu, ***ω***, *p<0.05 is significant.

**Figure 2:**
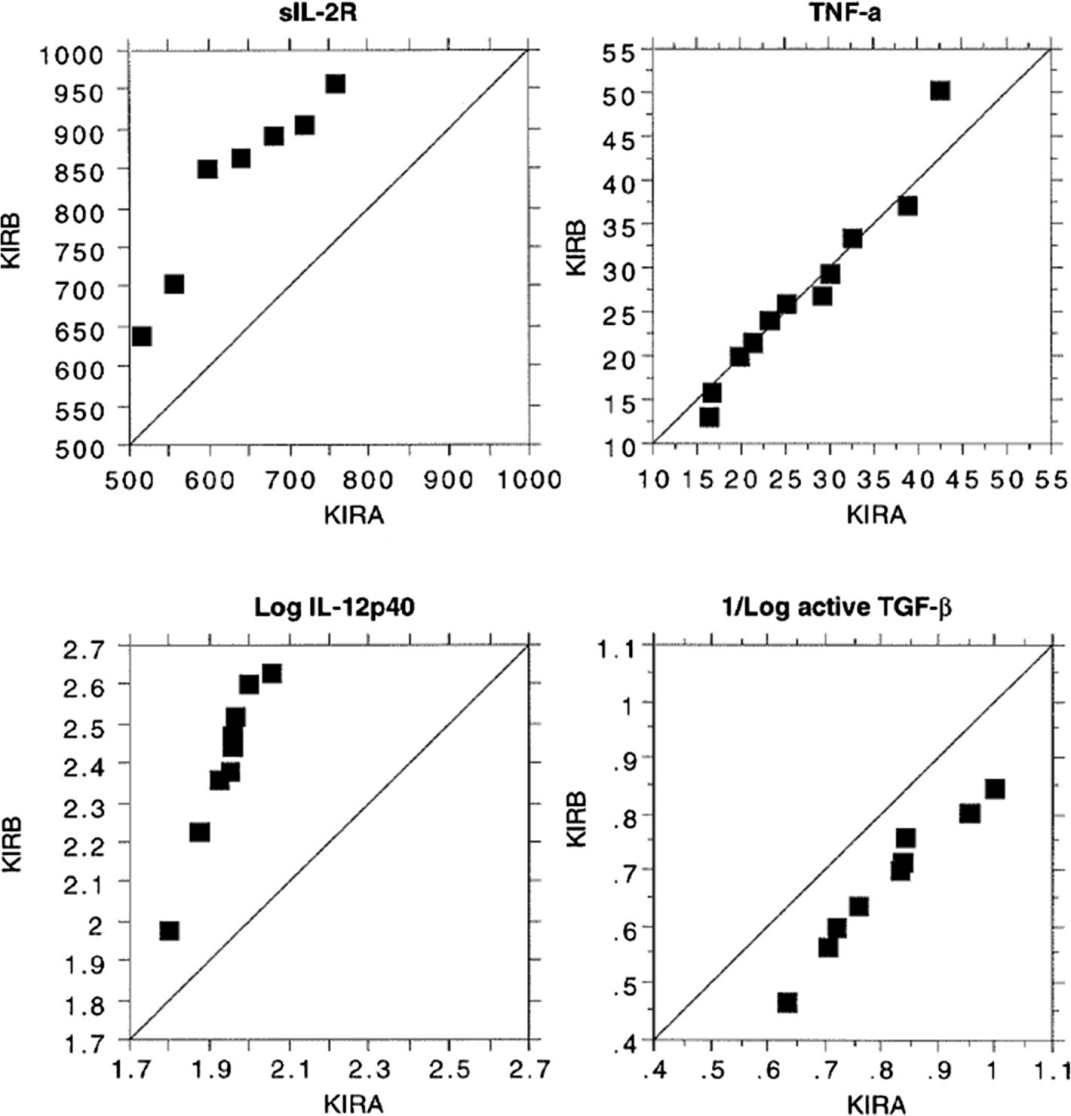
Percentile plots for Cytokines sIL-2R U/ml, TNF-α pg/ml, IL-12p40 pg/ml and log active TGF-β pg/ml for Octo/Nonagenarians from the Belfast Elderly Longitudinal Free-Living Ageing STudy (BELFAST) grouped by Killer Immunoglobulin Receptor Haplogroups A and B (KIR A and KIR B).

Percentile plots, for comparisons for KIR haplotype A v B percentiles for several cytokines, show a similar pattern with higher KIR B versus KIR A serum values for IL-12p40, similar values for TGF-α and lower values for TGF-β (Log active) (Figure 2).

Logistic regression was used to further explore whether carriage of the KIR A or KIR B haplotype by BELFAST octo/nonagenarians could predict serum cytokine level since logistic regression can compute using reduced subject numbers and does not depend on the normality or linearity of relationships. For IL-12, KIR B carriers were predicted to be likely to have higher cytokine levels compared to KIR A carriers by about 3% (OR 1.03, confidence limits CI 0.99–1.09; p=0.027) and TGF-β (active), a cytokine with an anti-inflammatory role, to have a 14% higher levels for KIR B compared to KIR A carriers (OR 1.14, confidence limits CI 0.99–1.09; p=0.002).

## Discussion

In this research we have analysed some of the relationships between NK cells and NK-related subsets, KIR A and B haplogroups and cytokines as they appear in subjects from the BELFAST study, who have evidence of being good quality survivors [26]. Here we demonstrate that KIR A haplotype carriers have significantly increased numbers and percentage of NK cells as a percentage of total lymphocyte count compared to KIR B haplotype carriers in BELFAST octo/nonagenarians. A similar non-significant trend emerged for male and female subjects. This means that KIR A haplotype octo/nonagenarians demonstrate a 60% higher NK cell numbers compared to B haplotype carriers, but with a similar width of distribution. Previously we had shown that NK cell numbers negatively associate with BMI in BELFAST octo/nonagenarians [27], in keeping with the original findings of Mariani et al. [28], and the more recent work of Lutz & Quinn [29]. The present analysis continues to demonstrate a negative association between BMI and NK cell numbers for this study cohort of BELFAST octo/nonagenarians irrespective of KIR haplogroup and also for KIR B haplotype carriers alone, though not for the smaller number of KIR A haplogroup carriers, probably related to lower statistical power. As suggested previously the apparent negative association between BMI and NK cell numbers may link through body fat percentage to BMI and physical exercise.

It is important to ask whether the increased NK and NK-associated subsets found in the context of apparently well elderly BELFAST 90 year olds represent evidence of robust good health and immune surveillance or are a response to chronic unrecognised illness. In support of enhanced immune surveillance, clinical and experimental evidence from animals demonstrated that increased NK number and activity links with improved immunity [30]. In man, while there is less accumulated evidence relating to NK cell numbers, an important 11-year follow-up epidemiologic survey showed an association between low NK cell activity and increased cancer risk [31] and clarified how important NK cell activity was in reducing tumour risk in adults [32]. There is also ongoing evidence of how effective an increased NK cell compartment is in the treatment of relapsed leukaemia [33]. Here the adoptive transfer of NK cells helps control the tumour burden. This has prompted interest in the development of innovative cancer therapies that are based on manipulating NK and NK-related subsets towards the treatment of solid tumour malignancies [34,35]. Conversely low or absent NK cells are associated with overwhelming infection particularly from viruses [36,37].

Increases in NK and NK-associated cells as noted in BELFAST octo/nonagenarians, have previously been reported in association with chronic infection or unrecognised illness [5,14,15]. Wikby, Pawelec and others in the Swedish NONA study have shown evidence linking long-lasting T CD8 cell clonopathies, with carriage of cytomegalovirus (CMV) infection and higher mortality [38-41] and this has been replicated more recently for carriers with high CMV seropositivity [42]. Increased mortality is associated with other chronic viraemias [43] and a recent paper associates all cause and cardiovascular mortality with levels of CMV seropositivity [44]. Pawelec and other researchers also reported that an inverted CD4/CD8 ratio was associated with chronic T cell clonopathies and poor outcome [38,40], though others including the BELFAST study authors [45] have found this to be either reversible or a non-consistent outcome in those followed up to the age of 100 years [39]. Viruses such as CMV or Epstein-Barr Virus (EBV), which have developed a largely commensal relationship with humans, block NK KIR detecting molecules by interfering with MHC class I expression so that their presence remains undetected and unchallenged [46,47], and many have adapted this strategy to perfection. Similarly down-regulation of MHC class I expression is a frequently observed phenomenon accompanying tumorogenesis [48]. Although a successful escape from adaptive immunity is possible, there is reason to believe that most evasive manoeuvers are counteracted early by NK cells [49]. If increased NK and NK-associated cells are related to chronic viraemia such as cytomegalovirus, it raises interesting questions about whether there could be therapeutic opportunities for improving health in older age groups [50].

We also examined the KIR haplotype percentages to look for the possibility of pleiotropic KIR effects and evidence for KIR gene frequency shift occurring in BELFAST subjects in association with their advanced quality ageing. In BELFAST octo/nonagenarians the KIR A genotype (AA) was present in 24% of elderly subjects compared with 76% carriage for KIR B genotype (AB+BB), with no male/ female predominance and these percentages are largely similar to those found in a younger cohort of geographically matched subjects [51,52]. This suggests that there has been relatively little reactive, pathogen-driven and age-related population immunological shift towards the more polymorphic and polygenic KIR B haplotype and visible KIR B frequency change. The distribution of A and B haplotype varies widely between distinct ethnic groups. The A and B haplotype frequencies are relatively constant in Caucasian populations [16,53]. However, across the world there is a spectrum of KIR A and B alleles; the A haplotype dominates in Korean, Japanese and Han Chinese populations with a frequency of approximately 75% [54,55] as compared to the Australian Aboriginal population where KIR A haplotype is around 13% with a shift to higher frequencies of KIR B haplotypes [23]. These differences may reflect both founder effects and pathogen-driven selection and seem likely to account for some of the world-wide variation in disease susceptibility. The differing global KIR A and B haplogroup frequencies suggests that the KIR B haplotype has been subject to more rapid diversification as a result of pathogen-mediated selection for KIR B genes which are greater in number and more polymorphic compared to the more limited gene set carried within the KIR A haplotype [17,56]. This concept would therefore suggest that BELFAST octo/nonagenarians do not or have not lived in a pathogen-challenged environment since B KIR B gene frequency does not demonstrate any major age-related significant shifts within the local population [57,58].

In the BELFAST octo/nonagenarians the most frequent KIR gene frequencies are KIR2DL1 (95%), KIR3DL1 (94%) and KIR2DL2 (88%). All these inhibitory KIRs belong to Haplogroup A and link with HLA Class I antigens in ligand specificity; KIR2DL1-HLA-C2; KIR3DL1-HLA-C1; KIR2DL2 -HLA-C1. The only activating gene in haplogroup A is KIR2DS4 which is also almost universally present in the BELFAST aged group (95%) and like the inhibitory KIRs, its ligand is within HLA-C. It was of interest to consider the KIR A and B haplogroups for the BELFAST octo/nonagenarians who have lived successfully into their 90s, since there is increasing evidence that co-associates of poor quality ageing such as auto-immune and chronic inflammatory disease, track with certain HLA-KIR haplogroup combinations [59,60]. The KIR A gene complex containing mostly inhibitory KIR genes tends to be associated with a lower risk of autoimmune diseases but shows higher risk of viral infections compared to KIR B haplotypes [60,61]. By example, KIR2DS1 and/or KIR2DS2 in the absence of HLA-C2 and HLA-C1 respectively, are associated with psoriatic arthritis, because of decreased potential for an associated inhibitory phenotype [62]. Conversely those homozygous for HLA-C2 lack ligand for KIR2DL1 and are associated with KIR2DS2 and diabetes [63]. Others have suggested associations for rheumatoid arthritis which have not always been replicated in different population groups [64-66]. Association with auto-immune disease may relate to the unknown character of the KIR B-ligand interactions, the controls and modulators of KIR-B activation activity and the ongoing effects on NK and NK-related cells. Similar frequencies of inhibitory type KIR A haplogroup as found for BELFAST octo/nonagenarians and for younger age groups seemingly support the concept of a relatively light burden of inflammation-related chronic age-related disease and this is also supported by BELFAST octo/nonagenarians phenotypic characteristics which show little evidence of diabetic or renal impairment and fit the descriptors of the ‘*Perl escaper*’ [26]. In early work on HLA-antigens in BELFAST nonagenarians, we reported an excess of the HLA-A1-B8-DR3 haplogroup (HLA*0100:Cw*0701:B*0801:DQA1) which associated with longevity in female BELFAST nonagenarians, and which we argued may relate to an activated immune system primed to manage effective infection and tumour surveillance but also contributing to female autoimmunity risk, if appropriately triggered [67]. Interestingly HLA-Cw7 homozygosity has been noted to affect the size of a subset of CD158+ NK cells [68] and Cw7 is considered to associate with the KIR2DL2 gene within the ligand HLA-C1. Although any associations are likely to be complex, we noted that this KIR2DL2 gene has a somewhat increased frequency in BELFAST nonagenarians (56%) compared younger local groups (49%) [51]. More recently an association has been described between the KIR2DS5 gene and protection from some age-related human diseases [69]. This finding is of interest in the context of good quality ageing and longevity, since this KIR gene shows a non-significant trend for higher frequencies for all BELFAST nonagenarians compared to younger groups (34% v 28%) and also for female nonagenarian compared to younger female subjects (38% v 27%), though these findings need replication in bigger studies.

Cytokines are produced by, and drive the NK and NK-related subsets in their cytotoxic stimulatory and inhibitory activities. IL-2 was the original cytokine first noted to accentuate NK cell activity [70] and we have previously shown an association between NK cell and related NK subsets and serum sIL-2R levels [24]. Like its sister pro-inflammatory cytokine IL-2, IL-12 and its heterodimer IL-12p40 associate with NK cells [71] which could be consistent with the original findings of their supportive role as factors promoting natural killer (NK) and cytotoxic T lymphocyte (CTL) activities [72,73]. The synergistic effect of combined IL-2 and IL-12 in promoting cytotoxic activity of NK cells through IFN-γ production, has been translated into a clinical therapeutic role for IL-12 in augmenting NK cytotoxity in cancer [74,75]. If replicated with larger study numbers, increased serum IL-12 levels associated with KIR B haplotype carriers in the current study, might suggest a potentially translatable outcome for KIR B subjects facing cancer-adjuvant therapy [76]. TGF-β, considered an immunosuppressive cytokine, reduces numbers of NK CD56(^dim^) cells [77,78], inhibits production of IFN-γ and TNF-α in vitro [79] and is now considered important in facilitating immune evasion in a cancer microenvironment through suppression of cytotoxic lymphocytes [80]. In the present study, although numbers are small, BELFAST nonagenarians who were KIR B haplotype carriers showed a trend for lower values for TGF-β. Conversely, TGF-β (active) values tended to be increased, which seems likely to be in keeping with its release from multiple tissue sites by proteolytic processes and offering differing roles.

Although the cytokine comparisons between KIR A and B haplogroups for BELFAST octo/nonagenarians must remain tentative because of small group numbers, the present analysis tends to shows accentuated cytokine profiles with the suggestion that KIR B haplotype carriers tend to produce higher amounts of the pro-inflammatory cytokines which could serve to heighten and prime the immune response. Normal cells are considered to express very few ligands for activating KIR receptors making it unlikely that the threshold for NK cell activation is reached under normal circumstances. A process of NK ‘*education*’ has been suggested to provide a diverse population of NK cells with different effector thresholds [81]. The question has therefore to be asked as to why there appears to be an activated cytokine milieu in BELFAST octo/nonagenarians and how this interacts and affects the immune effectiveness of the NK and associated NK-related cellular compartment. In a previous study we noted an association between NK cells and the CD3+CD69+ subset, one of the earliest signatures of immune activation [82]. In the BELFAST octo/nonagenarian group therefore, the NK cells appear primed for action, particularly for subjects who were KIR B haplotype carriers. Possible reasons for immunological up-regulation in the context of apparent good quality ageing could relate to early and superior cancer surveillance, response to low grade pathogens or a ‘*hormetic*’ response to a range of stressors and toxins related to imperfect gut, skin and endothelial barriers associated with increasing age.

There are several limitations to our study. First, our sample was screened for good health and this sample selection could have biased the outcomes since those with active disease at baseline were excluded. The ‘*elite elderly*’ subjects, when enlisted into the Belfast Elderly Longitudinal Free-living Ageing Study (BELFAST) project, were community-living, cognitively intact and met the criteria of the Senieur protocol, rather than representatives of an ‘*all comer*’ octo/nonagenarian cohort. They were consistent with the concept of the Perl ‘*escaper*’ or the 15% of elderly people who have reached the age of 90–100 with no overt signs of illness [1,26]. In addition the differing and reduced subject numbers, materials available and variables measured, could also have reduced the statistical power, particularly for male subjects and KIR A haplogroup carriers. Subject frailty, perceived or real vulnerability about lack of autonomy [83] are important and ongoing issues and make research in this group of subjects challenging and contribute to incomplete datasets. The findings in the BELFAST study should therefore be considered preliminary and exploratory and need to be replicated by other groups in other places with increased statistical power. However research studies with 90-year-olds remain scare but are highly important as this group is the fastest growing sector of the population and the subject group about whom we know relatively little medically, scientifically and economically.

Natural Killer cell populations, their KIR receptor complexes and associated cytokine profiles which both generate and drive their responsiveness, are highly effective collaborators in controlling, patrolling and protecting our immune landscape thorough out life. The intrinsic and extrinsic factors that shape human NK cell diversity remain incompletely understood. However, three factors appear to influence the structure and function of the KIR repertoire; KIR gene diversity especially haplogroup B; HLA class I -A, -B and -C ligands, and a program of sequential receptor acquisition during NK cell development which sets NK activation thresholds and could be related to the cytokine milieu. In recent research we have demonstrated that BELFAST octo/nonagenarians show maintained global methylation [84], supporting the concept of a competent immune system and it seems likely that hypo/hypermethylation of individual promoter genes provides another pathway through which lifestyle, nutrition, stress etc. modulate and change the activation/inhibitory patterns of KIR genes [85] and can fingerprint the ageing immune profile.

In the present research we have attempted to dissect out some elements of this immune landscape. We report some of the relationships between NK cells and NK-related subsets, KIR A and B haplogroups and cytokines as they appear in subjects from the BELFAST study. The findings across the 3 interacting domains should be considered exploratory but may serve to stimulate debate and encourage replication studies to improve our understanding about the immune signatures of those who live successfully into their 90s and who fit the criteria of the Perl ‘*escaper*’ model of successful ageing*.*

## Materials and methods

### Subjects

Ninety three unrelated consecutively enrolled subjects from the BELFAST study, age range 80–97 years, 70% female and 30% male were included in the elderly study sample. Elderly and very elderly subjects enlisted into the **B**elfast **E**lderly **L**ongitudinal **F**ree-living **A**geing **ST**udy (**BELFAST**) study gave written consent, were apparently well, lived independently in the community, were mentally competent [86] and met the criteria for inclusion in immuno-gerontological studies using the Senieur protocol [27,87]. Elderly subjects had a range of anthropometric measurements together with blood sampling carried out by a research nurse who visited at home as previously described [88,89]. Not all subjects provided adequate sample material for DNA separation, NK cell phenotyping and the full range of cytokines analyses. KIR gene characterisation was available for 77 subjects. All subjects gave written consent for inclusion in the study and permission for the study was given by Queens University Ethical Committee.

### Cell phenotyping and flow cytometric analysis

Natural Killer Cell (CD3-CD56+CD16+) and CD57+CD8+ subset analysis for elderly subjects from the BELFAST study only, was carried out as previously described [24]. Briefly, blood was collected into K_3_ EDTA, processed within 3 hours of collection and cells labelled in whole blood using the following two fluorochrome-labelled monoclonal antibody combinations: anti-CD3-FITC/CD16+CD56-PE and CD57-FITCE/CD8-PE (Becton Dickinson, UK). Flow cytometric analysis was performed on a FACScan instrument using Simulset software (BD). Routine haematological parameters were analysed on a Coulter STKS hemocytometer. Compensation settings and gates were established on negative controls. CD8/CD57 subsets were defined as CD8(^low^)+CD57+ and CD8(^high^)+CD57+ as previously described.

### Cytokine and receptor analysis

Matching BELFAST serum samples collected in endotoxin-free tubes and stored at −70°C were used for cytokine analyses. Cytokines were measured in batches from the same ELISA kits; IL-12 [IL-12p70 and Il-12p40], IL-10, TNF-α and TGF-β Genzyme; sIL-2R and sIL-6 Medgenix; IL-6 R&D Systems. Detection limit-6pg/ml for IL-10; 24U/ml for sIL-2R; total IL-12 <800 pg/ml; IL-12p70 <256 pg/ml; TGF-β, 0.75 pg/ml;TNF-α, 3 pg/mL; IL-6, 0.7 pg/mL.

### KIR genotyping by polymerase chain reaction (PCR)-sequence specific oligonucleotide probe (PCR-SSOP) hybridization protocol

Genomic DNA was extracted from the buffy coats of peripheral blood by the salting out method [90]. PCR amplification was performed using appropriate primer combinations and KIR genotypes, gene numbers and frequencies were identified and enumerated for the BELFAST subjects as previously described [51]. A KIR haplotype was identified as a fixed gene content of 4 inhibitory genes-KIR 2DL1, 2DL3, 3DL1 with 2DS4 as the single activating gene. Group-B KIR haplotypes were identified as containing variable gene combinations encoding more activating KIRs which did not include 2DS4. KIR AA haplogroup was designed KIR A and AB and BB haplogroups designated KIR B for analysis purposes.

### Statistical analysis

Results and statistical analysis for this study involved the octo/nonagenarian group from the BELFAST study categorised by KIR A and B haplotypes for NK and related subsets and pro and anti-inflammatory cytokines. For each variable, values are expressed as mean and standard deviation (SD) or as medians and range for non-normal distributions. Differences in subject characteristics, NK and related subsets and cytokine variables were categorised by KIR A or B haplotype status and analysed by students-test or Mann–Whitney U (mwu) as appropriate using Statview and SPSS version 16 programmes. Smaller datasets should be considered to provide descriptive information only. Logistic regression analysis and odds ratio (OR) was used to predict differences in KIR A and KIR B categories for those variables significantly different or close to significance by students t-test or mwu. P value <0.05 was used for nominal significance rather than Bonferroni correction being applied.

## Competing interests

The authors declare that they have no competing interests.

## Authors’ contribution

IMR set up the BELFAST study, provided biological materials, and planned the study, LDM, OAR, MDC, DM carried out the molecular genetics study, SEMcN, HDA carried out the cytokine analysis, IMR wrote first draft and all authors contributed to the manuscript. All authors read and approved the final manuscript.
